# Answering the call for values-based anti-doping education—An evidence-informed intervention for elite adolescent athletes in Germany and Austria

**DOI:** 10.3389/fspor.2022.859153

**Published:** 2022-09-23

**Authors:** Theresa Manges, Kevin Seidel, Nadja Walter, Thorsten Schüler, Anne-Marie Elbe

**Affiliations:** Department of Sport Psychology, Leipzig University, Leipzig, Germany

**Keywords:** anti-doping intervention, doping prevention, morality, values-based, empathy, moral disengagement, moral norms, anticipated guilt

## Abstract

Doping has serious negative consequences for athletes and the integrity of sports, implying the need for effective prevention programs. Since educating young athletes about doping-related knowledge is deemed to be not sufficiently effective to minimize doping, a focus on values, emotions and morality is seen as a promising approach and previous research indicates which variables exactly could be addressed in anti-doping efforts. These variables are anticipated guilt, empathy, moral disengagement, and collective moral norms, since these constructs have been strongly and consistently linked to doping intention, likelihood, or behavior. Therefore, the purpose of this study was to develop a values-based anti-doping intervention, which targets the aforementioned variables, and to evaluate its effectiveness in producing changes in outcomes in comparison to an information-based intervention and a waiting control group. To evaluate their effectiveness, both interventions, which each consist of six 45-min sessions (one session per week) were implemented in a sample of 321 young elite athletes, aged 13–19 years, from a broad range of team and individual sports. Thirty different teams, training groups or classes were randomly assigned to either the values-based intervention, the information-based intervention, or to a waiting control group. Doping intention, doping susceptibility as well as the above mentioned variables were assessed at pre- and posttest and, for participants of the values- and information-based conditions also at a 3 to 4-month follow up. Within a multilevel modeling framework general linear mixed regression analyses revealed that the values-based intervention, compared to the control group, was able to decrease athletes' moral disengagement and increase their anticipated guilt immediately after the intervention (at posttest), whereas no effects for the information-based intervention emerged. Looking at how the outcomes developed over time (i.e., at the follow up measurement), it could be demonstrated that the reduction in moral disengagement sustained. The increase in anticipated guilt, however, was not sustainable and, surprisingly, decreased from post to follow up. Furthermore, athletes in the values-based intervention reported higher empathy from post to follow up, which could possibly indicate a “delayed” effect. This study provides support that a values-based approach can produce changes in some, yet, not all addressed variables and specific elements from this intervention could potentially be a useful addition to traditional anti-doping education (i.e., information provision).

## Introduction

Even though sport is an ideal setting to foster moral competencies, these competencies are at the same time challenged by one of sport's major threats, namely doping. As defined by the World Anti-Doping Agency (WADA), doping comprises the occurrence of one or more out of 11 anti-doping rule violations set out in the World Anti-Doping Code (1). To name a few, evading or refusing sample collection, possessing and (attempted) trafficking prohibited substances or methods, or (attempted) tampering of a doping control all belong to anti-doping rule violations. However, the most common violation is the use or attempted use of banned substances or methods to enhance performance. With estimated prevalence rates ranging from 14 to 39% (2) up to 44–57% (3), it is obvious that doping is a widespread problem, especially in elite sport. Due to the potential health damages of doping, its threat to fair play and its negative influence on the integrity of sports, there are considerable efforts to minimize doping. The most commonly used strategy so far has been the detection and deterrence approach (4), which assumes that detecting and sanctioning doping will deter other athletes from resorting to it. However, since this approach is costly and at the same time not sufficiently effective in reducing the prevalence of doping, an educational anti-doping approach is viewed as promising and has been gaining importance [see (5)]. WADA [(6), p. 56] recognizes education “no longer [as] […] a worthy but optional extra”, but as “an essential and central pillar of the global anti-doping program”. This statement is further supported by the publication of the International Standard for Education [ISE, (7)] in which WADA also emphasizes the need for developing and delivering doping prevention programs that go beyond raising awareness and providing information about doping and that focus on a values-based educational approach. The purpose of the present study is to address that need and to develop, deliver and evaluate a values-based doping prevention program.

### Current state of anti-doping programs

Having a closer look into the overall “prevention through education” approach, one can typically differentiate between “information programs” and “education programs” (5, 8). In addition, the ISE (7) distinguishes between a cognitive and an affective domain. Information programs align with the cognitive domain and aim at creating awareness and increasing knowledge about doping (e.g., forbidden substances, side effects, consequences) in athletes, coaches and other support personnel in order to prevent -especially unintentional- doping (8). Education programs that align with the affective domain are meant to go beyond a knowledge transfer and often focus on “values-based” education (9). According to the ISE, values-based education means “delivering activities that emphasize the development of an individual's personal values and principles. It builds the learner's capacity to make decisions to behave ethically” [(7), p. 10]. As such, values-based anti-doping education can address emotions, motives, attitudes and values and often incorporates the fostering of moral competencies. This seems plausible since a person's morality, defined as his or her beliefs and practices about what is right or wrong, which is developed over time, influenced by social contexts (i.e., group, culture, society) and guided by personal values, is presumed to be shapeable/trainable [e.g., (10, 11)]. The following paragraph provides an overview of the current state of interventions/programs.

Prominent examples of information-based interventions are the ATLAS [Adolescents Training and Learning to Avoid Steroids (12)] and ATHENA [Athletes Targeting Healthy Exercise and Nutrition Alternatives (13)] programs, which both impart knowledge about a variety of unhealthy behaviors including doping. Research evaluating these programs in large samples of athletes did not demonstrate a significant decrease in the number of reported doping cases over a season or school year compared to a control group (14, 15) and showed only slightly decreased doping intentions compared to a control group (14, 16). Nevertheless, these two programs formed the starting point for a prevention initiative during the last two decades, marked by the development and evaluation of several anti-doping interventions. A recent example for interventions that focus on knowledge about doping and particularly about the associated health consequences is WADA's Athlete Learning Program about Health and Anti-Doping (ALPHA); however, while Murofushi et al. (17) found the ALPHA program to be effective in increasing knowledge about doping, they did not measure or report any outcomes on doping intentions or behavior.

Three recent reviews examining (a) 30 doping prevention studies (9), (b) 53 national anti-doping organizations' prevention campaigns (18), and (c) 14 anti-doping interventions (19) demonstrate that information-based (i.e., conveying anti-doping knowledge inclusive health consequences) approaches dominate other approaches (e.g., values-based approach). This is surprising, since the information-based approach has been criticized and deemed to have no or only a modest effect on reducing doping intentions and behavior (20, 21). Moreover, as the review results further indicate, information-only programs are rated significantly lower in usefulness and trust by athletes, compared to other, more comprehensive educational programs [e.g., (18)]. This emphasizes the need for multifaceted education in which at least one additional approach is implemented together with the information-based approach.

As a response to the latter, there is a trend towards including additional elements beyond mere information provision, and these additional elements often focus on targeting psychological variables that are empirically related to doping proxies (e.g., doping intentions, doping likelihood). An example is the Hercules program (22) which conveys knowledge to athletes in combination with providing information on the ethics of doping and on resisting peer pressure to dope. Other programs also include psychological, moral and ethical aspects of doping such as fair play and the values of sport (23–25), alongside knowledge transfer. The iPlayClean program (26) addresses a variety of topics such as doping myths, health, nutritional supplements but also motivation, playing fair and resisting temptations and was successful in reducing favorable attitudes toward doping in adolescent athletes. A somewhat different approach is a media literacy intervention, which deals with the moral aspects of doping and how the media may disregard them (27, 28). Noteworthy is also a program that focusses on coach education and which showed that athletes were less willing to take banned substances when their coaches adopted a motivationally supportive communication style in regard to doping-related discussions [CoachMADE (29)].

Looking at the interventions that exclusively focus on psychological, moral and ethical aspects {which, for the sake of this study and in line with the distinction pointed out above [see (8) and ISE; (7)], will be labeled as “values-based” programs}, three programs stand out. Based on the Konstanz dilemma-discussion method (10, 11) Elbe and Brand (30) developed an ethical decision making training for young athletes. An online intervention with doping-specific moral dilemmas aimed at promoting moral reasoning in athletes. Although, contrary to the authors' expectations, this intervention slightly increased athletes' doping attitudes, the authors argue that the training challenged stereotypes in reasoning about doping. Most recently, the research of Kavussanu et al. (31, 32) showed that intervening specifically on moral and psychological variables, which are associated with doping behavior proxies in empirical research, seems to be a promising way to prevent athletes from doping. For both studies, adolescent athletes from the UK and Greece were recruited and randomly assigned to an intervention or control group. In study one (31) the researchers developed a moral intervention that addressed moral identity, moral disengagement, and moral atmosphere and compared it to a so called “standard”, i.e., information-based intervention that conveyed knowledge about the health consequences of banned substances, the risks of nutritional supplements, the doping control process, etc. In study two (32) a psychological intervention which targeted anticipated guilt, moral disengagement, and self-regulatory efficacy was developed and, likewise, compared to the information-based intervention. Results showed that, in study one, both the moral and information-based intervention were able to reduce doping likelihood at a post measure as well as at a 6-month follow-up. Study two revealed that the psychological intervention was superior to the information-based intervention in reducing doping likelihood from pre to post, but the sustaining effects at the follow-up were similar in both intervention groups. However, as both studies lacked a no-intervention waiting control group, it is not entirely clear if the changes in outcomes were caused solely by the contents of the interventions. Incorporating a no-intervention waiting control group into these designs would help provide an untreated comparison for both active intervention groups.

The current state of anti-doping interventions suggests that intervention programs targeting psychological and moral doping-related variables can have a meaningful impact on athletes' decision to dope. Over the next paragraphs we address the question which specific variables should be targeted in an intervention.

### Theoretical and empirical background

In general, research indicates that both personal moral variables such as moral disengagement and social context variables such as perceived norms should be taken into account (33, 34). This is in line with Bandura's (35) social cognitive theory of moral thought and action which has served as a foundation for recent anti-doping research [e.g., (32, 36)] and also informs the study at hand. According to Bandura (35), a person's behavior is governed by the moral standards that this person has developed and internalized through socialization processes. When engaging in a behavior that contradicts one's own moral standards people usually experience negative emotions such as guilt or shame. In order to avoid such affective self-sanctions people tend to behave according to what is expected based on their moral standards. Those emotional reactions, positive or negative, play a central role in regulating moral conduct since they operate anticipatorily.

The role of anticipated guilt, a regulatory moral emotion, has been investigated increasingly in relation to transgressive behavior and especially doping over the past years. Studies outside the sport context show that the proneness or anticipation to feel guilt has been inversely associated with bullying behavior and aggression (37, 38). Doping specific research from a wide range of sports evidences a strong inverse relationship between athletes' anticipated guilt and doping likelihood/doping intentions [e.g., (36, 39–41)]. Athletes know that by doping they would transgress the rules of sport and therefore engage in cheating behavior which is seen as morally wrong (42). Hence, they may anticipate feeling unpleasant emotions like guilt or regret when making the decision to dope. Since people try to avoid such feelings, anticipating those unpleasant affective reactions can therefore deter athletes from doping.

However, people are able to situationally disengage from their moral standards in order to minimize the expected negative emotions that would typically arise from transgressive conduct like doping. As explained by Bandura (35), people do so by making use of one or more cognitive mechanisms, collectively termed moral disengagement (MD). Past research identified six mechanisms relevant to doping in sport (43). In a doping-specific situation athletes may, for instance, try to justify their behavior by arguing that “everyone on the team is doping, too” (i.e., diffusion of responsibility) or that the coach, teammates or support personnel pressured them to dope (i.e., displacement of responsibility). Also, athletes may use favorable names for doping substances or methods, such as “vitamins” or “medical treatment”, making doping sound less severe (i.e., euphemistic labeling). Further mechanisms are downplaying or ignoring the harmful consequences by saying that it “does not really hurt anyone” (i.e., distortion of consequences) or by comparing doping to transgressive behaviors from other contexts that seem worse, such as criminal conduct (i.e., advantageous comparison). Finally, athletes may cognitively restructure their transgressive behavior into a “good” behavior by justifying, for example, that doping “helps the team”, thereby making it appear acceptable (i.e., moral justification). The use of the described mechanisms facilitates athletes' doping behavior through minimizing the negative feelings typically associated with it. Qualitative research highlights the importance of MD in regard to doping [e.g., (44)] and quantitative designs support the strong and positive relationship between MD and doping intentions, doping likelihood and reported doping behavior [e.g., (33, 36, 39, 43, 45, 46)].

Another key aspect of the social cognitive theory of moral thought and action, which is important in relation to doping is empathy (36). Empathy reflects the capacity of vicariously producing emotional and cognitive responses to another person's emotional state (47). That means, an empathic person possesses the ability to change the perspective and see the world from another person's view. It is supposed that, the better an athlete can understand the consequences of his or her unethical behavior for others, the more difficult it is to engage in such behavior (35). Although empathy has received less research interest, there is empirical evidence that empathy is negatively linked to antisocial behavior in sport (48, 49). Furthermore, Boardley et al. (36) demonstrated a negative predictive effect of empathy on MD and a positive predictive effect of empathy on anticipated guilt, thereby suggesting that increased empathy is associated with lower MD, higher anticipated guilt and hence, with reduced doping behavior or its proxies. These findings concerning the interplay between guilt, empathy and MD are consistent with theory (35) and with empirical evidence from other contexts, such as the business context (50), making all three variables promising targets in our intervention.

Nevertheless, not only personal moral variables play a role in explaining doping behavior but also social context variables should be considered, as suggested in various doping research models [e.g., the Sport Drug Control Model (51); or the Life Cycle Model (52)] and underpinned by the social cognitive theory (35). Since an athlete's social context is determined primarily by his or her team or training group, collective group norms and values, that develop over time through the interaction of group members and define what kinds of behaviors are considered acceptable within a group, are of interest. Sport research shows that collective group norms, sometimes referred to as moral atmosphere [e.g., (39)] moral climate [e.g., (53)] or collective moral attitude [e.g., (54)] strongly influence the moral behavior of group members. Doping research, specifically, provides evidence for a strong relationship between collective moral norms and doping intentions (33, 34, 54). In light of these findings, integrating and targeting collective moral norms/attitudes may be beneficial in our doping prevention program.

After having examined the theoretical and empirical background on which target components should be incorporated in our intervention, the question of the timing of doping prevention efforts is a crucial point as well (7), that is, which target group the intervention should be designed for. WADA (7) points out that anti-doping efforts should occur at an early phase of athletes' careers, so that their first contact with anti-doping happens through education rather than doping control procedures. Researchers agree on that by stating that primary prevention before potential onset of doping behavior is beneficial (20, 55). Asking the athletes themselves, qualitative studies provided additional support for an early implementation of anti-doping programs (56, 57). Adolescence is seen as a critical doping entry phase (9) but at the same time as a phase paramount for developing and changing values, attitudes and moral behavior (58, 59). Therefore the intervention program was designed for adolescent elite athletes.

### Aims of the present study

As WADA (6, 7) declares education a central pillar in the global fight against doping, the need for effective education programs is obvious. A focus on a values-based educational approach is emphasized in the ISE (7) and has become increasingly present in anti-doping efforts. Some intervention studies have gone beyond the knowledge transfer and have included elements that target psychological and/or moral variables that are empirically associated with doping [e.g., (23, 24, 28)], yet only a few were able to show long-term effects in reducing doping proxies, such as doping likelihood (31, 32), doping susceptibility and attitudes (26), highlighting the importance of study designs that evaluate long-term effectiveness.

Concerning the theoretical and empirical background on doping-related psychological and moral factors, several key variables from Bandura's social cognitive theory of moral thought and action (35) have been found to strongly relate to doping likelihood, intention or susceptibility [e.g., (36, 39)] and therefore should be considered in our intervention program (i.e., anticipated guilt, moral disengagement, empathy and collective moral norms). When designing this intervention we could also draw upon previous studies by Elbe and Brand (30) as well as Kavussanu et al. (34) which served as inspiration.

In conclusion one can state that there is knowledge about the effectiveness of doping prevention programs, about the variables that should be addressed and about the most promising target group, namely adolescent (elite) athletes. However, most of this research was conducted in countries outside the German speaking countries [with exception of the study by Elbe and Brand (30)].

Consequently, the present study aimed at, first, developing a values-based intervention program, designed for adolescent athletes and based on theory and empirical evidence; second, implementing the intervention program in young elite athletes in Germany and Austria along with an information-based program; and, third, evaluating its effectiveness. For the sake of the latter we formulated two research questions, namely: (1) Are the values-based intervention and the information-based intervention effective in producing changes in outcomes after the intervention, compared to a waiting control group? and (2) Can changes in outcomes within each intervention group be maintained over time, i.e., at a 3 to 4-month follow up? The primary outcomes are doping intention and doping susceptibility and the secondary outcomes are anticipated guilt, moral disengagement, empathy as well as collective moral attitude/norms.

## Materials and methods

### Development of the intervention programs

The main purpose of the study's first stage was the development of the values-based intervention program, along with the development of an information-based program for reference purposes during the evaluation process. Both interventions were designed to be implemented by a facilitator/instructor in small groups of athletes (5–15 participants). They consist of six sessions, respectively, with each session lasting 45 minutes in order to enable potential realization within the timeframe of a school lesson. By having one session with one topic per week over a 6-week period, time for reflection and retention in between the sessions was warranted. This also followed previous findings that effective interventions should run over longer periods [2–10 weeks; (60)]. During the development it was key for us to base it on a high-quality pedagogical foundation and to ensure an appealing content delivery. Therefore, two experienced high school PE and ethics^1^ teachers, whose competencies and credentials were known to the research team members, were recruited to work for the project through a professional fee agreement and substantially contributed to the intervention development. Both interventions were grounded on a problem-based learning approach, which allows the learner to become actively engaged with the subject matter (61). For this purpose, fictional and real athletes' cases were included in the learning materials as the story-like character is seen to enhance immersiveness and makes the topic more approachable than talking about doping and related emotions, attitudes, etc. at an abstract level. Furthermore, to promote athletes' engagement, role-playing games and quizzes were applied; interaction and group discussions were encouraged; arguments were generated and contents, like posters or letters, were created. This approach aims at ensuring that participants effectively acquire skills and knowledge, reflect on the content and apply it to problem-solving in potential real-life situations (62). The first version of the two programs were presented to a number of coaches, teachers and stakeholders in sports within a project meeting. Minor content adjustments were made following the feedback received during the meeting (e.g., since students may need more time than originally allotted to comprehend and conduct the tasks/activities, two sessions were deemed as too full and activities were reduced to better fit the 45 min session length).

The delivery mode of the interventions was initially planned as face-to-face teaching and both interventions were pilot tested with soccer and judo athletes (in total 40 athletes, aged 15–18) in order to improve the interventions following the participants' extensive qualitative feedback. However, due to the pandemic situation, face-to-face teaching was no longer feasible and the teaching materials were adapted to an online format. During this adaptation process the content remained unchanged but the didactical methods had to be modified to digital synchronous teaching. For each session, the group of athletes comes together with the facilitator at a set time *via* the video communication platform Zoom and synchronously participates in the session. Special features of Zoom, such as breakout rooms and surveys but also further online tools, such as quiz-apps and a digital whiteboard software provided options for working together in small subgroups, creating mind maps, conducting opinion polls or visualizing information. The newly designed online materials were then tested in a further pilot study within the framework of the final theses of two trainee PE teachers in a sample of 10 handball athletes, aged 13–15. Participants in the pilot study provided comprehensive feedback as they were asked to fill in a feedback questionnaire after each session and to engage in a general feedback discussion after completion of the program. Both elements (i.e., each session's questionnaire and general discussion) addressed a wide range of issues, such as the sessions' content, comprehensibility, didactical methods, (technical) feasibility and timing as well as participants' engagement and commitment. Based on the athletes' feedback and the facilitators' impression, final, minor adjustments, especially regarding the timing, were made. An overview of both online intervention programs are presented hereafter^2^.

#### Values-based intervention

The values-based intervention was designed to encourage cognitive, emotional and group dynamic processes and to create awareness and reflection of personal values and attitudes. Grounded on theory (35) and empirical evidence [e.g., (33, 39)] and underpinned by previous intervention studies in and outside the German language area (30–32) the intervention aimed at affecting anticipated guilt, empathy, moral disengagement and collective moral norms. Each variable is addressed in one specific session (session 2–5), whereas session 1 and 6 form the intervention's framework.

*Session one* serves as an introduction to the topic “Doping—yes or no?” After words of welcome and a warm-up participants are asked to mark a spot in a coordinate system in a web-based interactive whiteboard, indicating if they generally approve or disapprove doping and if this was an easy or tough decision for them to make. Then, a doping-specific hypothetical scenario is presented *via* an animated video, depicting the moral dilemma situation of a young female athlete (“Lisa”) who wants to perform clean but now thinks about taking banned performance-enhancing substances as she witnesses her better-performing teammates talking about their substance-use. Working with such dilemma situations, based on the Konstanz dilemma-discussion method (10, 11), is seen as a beneficial method to confront athletes with, and train potential future decisions in their athletic career (63). The employed moral dilemma story originates from Elbe and Brand's (30) ethical decision making training and serves as a common thread through subsequent sessions. Once participants have seen the video, they are asked to imagine that they were in Lisa's situation and, again, enter their position in the coordinate system from Lisa's perspective. By comparing the before-and-after marks in the coordinate system and discussing related arguments for and against doping, the athletes should recognize the conflicts young athletes may face, when they encounter contradicting interests and values. The overall aim of this session is to encourage participants' reflection and to make them aware that decision making might be more difficult as soon as the situation becomes less hypothetical and more specific by describing an athlete's real dilemma.

*Session two* addresses anticipated guilt and refers back to Lisa's dilemma story. An animated video is presented, illustrating how the athlete now has decided to try the forbidden substances in order to keep up with her teammates. Participants are requested to create a fictional ending for Lisa's story. They have the option to end the story in two ways, either Lisa is caught doping or not. Regardless of the ending they choose, they are instructed to consider a variety of potential consequences and to pay special attention to feelings, the person in question might experience, thereby getting a glimpse of emotions such as guilt or regret. In the discussion that follows, some of the created stories are presented and the possible endings are visualized through an interactive whiteboard. The consequences the participants mention are clustered into categories like health, legal, financial, psychological, and social, with a focus on the latter two. Based on evidence from previous interventions (31, 32) the purpose of this session is to sensitize athletes about potential feelings of guilt and remorse that might occur when deciding to dope.

*Session three* targets empathy and tries to foster participants' ability to take the perspective of other persons that are potentially affected by one's doping behavior. The last part of the Lisa-dilemma-video is shown, which illustrates how Lisa has been tested positive in a doping control. Various characters, such as a former competitor, who has been awarded her medal 5 years later due to Lisa's doping, Lisa's parents, or a long-standing fan of hers, are introduced by the facilitator. The participants receive the task to write a diary entry from the respective person's point of view, addressing their feelings and thoughts. In Zoom breakout rooms, athletes who have been assigned the same characters come together to discuss their diary entries before presenting some of them to the entire group and reflecting on the feelings of those third persons that were involuntary affected by Lisa's decision to dope. In addition to training participants' perspective taking this session also highlights the tremendous consequences the decision to dope has for others. Thus, session three at the same time opens the way for session four, as it challenges the moral disengagement mechanism of distorting the consequences one's decisions have.

*Session four* deals with the construct of moral disengagement. After a brief definition athletes are encouraged to think about possible “justifications” a person who decides to dope could use. In order to learn about the different types of MD, participants take part in a memory game that has been prepared in the interactive whiteboard and are asked to connect real athletes' statements concerning their doping to the associated MD mechanisms. Then, an interview-video of a well-known athlete who doped is shown. Participants are asked to pay attention to the justification strategies this athlete used. They receive the task to prepare a role-play in groups, in which a journalist interviews a sportsperson who has been caught doping and several role-plays are presented and discussed at the end of this unit. Through this session's activities, participating athletes are expected to not only detect typical justifications for doping but also to challenge these by finding counterarguments. This process is especially fostered through the role-play (i.e., the journalist's role).

*Session five* targets the participating group's collective moral norms. The athletes get to know the values of sport by, initially, watching a video of an athlete who talks about the values of his clean-competing team. After discussing the video, the athletes, as a group, are invited to pick values from a catalog of values in the interactive whiteboard and sort them according to their perceived significance. This task is followed by finding arguments whether their top three values are reconcilable with doping behavior. This session's purpose is to provoke thinking about and reflecting on collective values and norms and to guide the group toward a shared understanding that doping is incompatible with the majority of sport values. Additionally, due to the fact that the athletes take part in the intervention as a group, it is assumed that all the sessions will impact not only their individual but also their collective moral norms.

*Session six* serves as a summary unit of all topics discussed in session one to five. Participants are assigned to two groups (in two breakout rooms) and instructed to apply their knowledge/skills to a new dilemma situation. Through analyzing the pros and cons, considering possible consequences for the athlete in question as well as for third parties that may be affected by this athlete's decision to dope, participants are expected to arrive at a sound decision. The two groups then present their respective dilemma stories, along with their analyses and final decision. This is followed by the instructor's summary, conclusion and fare-well.

#### Information-based intervention

The information-based intervention which served as a comparison was designed based on the German National Anti-Doping Agency's (NADA) current prevention program “Gemeinsam gegen Doping” (“Together against Doping”) and strictly followed its content but employed various didactical methods to create an intervention that is comparable to the values-based intervention regarding its engagement enhancing delivery and interactive character. In collaboration with the NADA and the two ethics and PE teachers, six sessions with the following topics were developed:

*Session one* Introduction. Doping—what is it?: Through providing a worksheet about doping and conducting breakout rooms for exchanging ideas before coming back together in the plenum, students elaborate a definition of doping, discuss the role of NADA and WADA and are introduced to famous doping cases and frequently asked questions about doping;

*Session two* The prohibited list: Through examples of famous doping cases prohibited substances and methods as well as their side effects are discussed. Acquired knowledge is deepened by connecting substances and methods with their respective effects and side effects within a digital whiteboard based memory game and by editing a “truth or lie”-text on that topic.

*Session three* Consequences of doping: Again, stories of real athletes are utilized in order to make participants understand the consequences doping can have on different levels. Participants are asked to read stories about persons who doped, complete a worksheet listing the consequences, and, finally categorize these into legal, social, health related and financial consequences.

*Session four* Doping control procedure: After an introduction exercise “Position yourself regarding the statement that doping controls are manipulated anyways” athletes watch a NADA video, illustrating the procedure of an urine doping control. Thereafter athletes have to sort terms/words in the correct order of the procedure's steps on a digital worksheet (e.g., “A and B sample” goes to step 4 “Packaging of the sample”). The aim of this session is to convey information about doping control procedures thereby emphasizing that the statement in the beginning of the session is not correct.

*Session five* Supplements and related risks: Athletes are provided with a digital mind map in which comprehensive information about nutritional supplements, promised effects and related risks are presented before they engage in a quiz, prepared with the online tool *Kahoot*, whereby they can test their knowledge and compete against each other.

*Session six* Summary and internet resources: In small groups *via* breakout rooms students prepare a creative poster (in Microsoft Word or Power Point or other tools) which should contain all information and facts that they remember from the preceding sessions. After working on these together for a few minutes they are allowed to use anti-doping internet resources, e.g., NADA and WADA website and other important websites [e.g., Anti-Doping Administration and Management System (ADAMS), Cologne list] in order to complete and improve their posters, which are finally presented in the plenum and commented (and corrected if needed) by the instructor.

### Implementation and evaluation

#### Design

The study was carried out as a cluster randomized controlled trial (RCT), in which clusters of athletes (i.e., teams/training groups/school classes) were randomly assigned to the different conditions. The RCT was delivered in the sport school- and sport club setting in Germany and Austria.

The first research question was addressed by implementing a three-arm parallel-group trial with two measurement points, with group/condition (values-based, info-based, control) as between-subjects factor and time (pre, post) as within-subjects factor.

Research question two was investigated by collecting participants' data (values-based and info-based condition) at a third measurement point, thereby employing a two-arm parallel-group trial with three measurement points, whereby group/condition (values-based, info-based) was the between-subjects factor and time (pre, post, follow up) was the within-subjects factor.

#### Participants

A total sample of 321 athletes from 30 teams, classes, or training groups were recruited. Their demographic characteristics are presented in detail in Table 1. Inclusion criteria were performing a team- or individual sport on competitive level and being aged between 13 and 19 years old. Based on comparable intervention studies [e.g., (26, 32)] we specified an effect size of 0.45, set alpha at 5% and aimed for a power of 0.80. Using Optimal Design Software (64) for cluster RCT with outcomes on the person-level, it was shown that for the primary outcome of doping intention a sample of 30 clusters with on average 11 athletes per cluster provided the envisaged power of 80% to detect a moderate effect size of 0.45, accounting for an intraclass correlation coefficient (ICC) of 0.10.

**Table 1:** Participant demographics.

		**Values**	**Information**	**Control**
		***k* = 14, *n* = 134**	***k* = 9, *n* = 114**	***k* = 7*, n* = 73**
Age	Mean (SD)	15.59 (1.54)	15.38 (1.67)	15.15 (1.60)
Gender *n* (%)	Female	56 (41.8%)	38 (33.3%)	47 (64.4%)
	Male	77 (57.5%)	75 (65.8%)	26 (35.6%)
	Other	1 (0.7%)	1 (0.9%)	
Sport type *n* (%)	Team	30 (22.4%)	45 (39.5%)	38 (52.1%)
	Individual	104 (77.6%)	69 (60.5%)	35 (47.9%)
Competition level *n* (%)	Regional	32 (23.9%)	26 (22.8%)	39 (53.4%)
	National	68 (50.7%)	75 (65.8%)	31 (42.5%)
	International	32 (23.9%)	12 (10.5%)	2 (2.7%)
	Other	2 (1.5%)	1 (0.9%)	1 (1.4%)
Years main sports	Mean (SD)	7.06 (2.94)	8.95 (2.88)	7.55 (2.75)
Hours/week training	Mean (SD)	13.72 (4.85)	12.11 (5.58)	13.69 (4.20)
Doping prevention measure before *n* (%)	Yes	52 (38.8%)	28 (24.8%)	29 (39.7%)
	No	82 (61.2%)	85 (75.2%)	44 (60.3%)

*k* = 30 teams/classes/training groups, *N* = 321 athletes.

#### Measures

##### Doping susceptibility

Doping susceptibility which reflects the “absence of a firm resolve not to engage in doping activities or to give any consideration at all to an offer to do so” [(65), p. 481] was measured with the item “If you were offered a banned performance-enhancing substance under medical supervision at low or no financial cost and the banned performance-enhancing substance could make a significant difference to your performance and was currently not detectable. How much consideration would you give to the offer?” utilizing a 7-point Likert-type scale (1 = none at all; 7 = a lot of consideration). Participants responding with “none at all” are seen as non-susceptible, whereas all other answers would express that the respondent can be classified as susceptible (65). This one-item measure has been used in previous studies and has been found to be a suitable instrument for indicating doping susceptibility (65, 66).

##### Doping intention

Our primary outcome doping intention was ascertained *via* two scenarios, developed and used by Kavussanu et al. [e.g., (31, 39, 43, 67)] and translated within a previous study (54). These scenarios describe hypothetical situations in which athletes are tempted to dope in order to enhance their performance (scenario 1) or to recover faster from an injury (scenario 2). As described in Kavussanu et al. (67), after each scenario, athletes have to rate the following three questions “How likely…/How tempted…/How willing would you be to use the banned substance?” on a 7-point Likert-type scale (1 = not at all likely/…tempted/…willing; 7 = very likely/…tempted/…willing). The mean of the six items was computed for a total score of doping intention, whereby higher scores represent a higher doping intention. With the help of these scenarios a vicarious behavioral intent and thereby answers that are more truthful can be obtained, as compared to a direct question if one intends to dope (68). Manges et al. (54) proves very good internal consistency (α = 0.88) for the German version of doping intention.

##### Moral disengagement

A German version of the Moral Disengagement in Doping Scale (43) was employed to measure doping moral disengagement. Participants rate their degree of consent with six statements on a 7-point Likert-type scale (1 = strongly disagree; 7 = strongly agree). Items are, for instance, “Doping does not really hurt anyone” or “Doping is just a way to maximize your potential”. The mean of the six items was computed as a total moral disengagement score with higher scores implying greater moral disengagement. With values of α = 0.69 for internal consistency and *corrr*_tt_ = 0.80 for split-half reliability as well as demonstrated construct validity the German version represents an appropriate measure of doping moral disengagement (69).

##### Anticipated guilt

To assess anticipated guilt, we used the guilt subscale of the State Shame and Guilt Scale (70). Participants are requested to answer five items with the preceding stem “If I had used a banned substance…”. Example items are “I would feel bad about what I had done” or “I would feel remorse, regret”. Answers were given on a 7-point Likert-type scale (1 = not at all; 7 = very strongly). For a total score of anticipated guilt the mean over the 5 items was computed; the higher this score is, the higher is the degree of anticipated guilt. Evidence of this measure's internal consistency (α = 0.82) is reported by Marshall et al. (70) and preliminary analyses preceding this study also reveal very good internal consistency (α = 0.92) for the translated German version.

##### Empathy

For the assessment of empathy, two subscales of the German version, i.e., “Saarbrücker Persönlichkeitsfragebogen” [IRI-S-D; (71)] of the Interpersonal Reactivity Index (72) were utilized. The respective four items of the subscales empathic concern (example item “I am often quite touched by the things that I see happen”) and perspective taking (example item “Before criticizing somebody, I try to imagine how I would feel if I were in their place”) were answered on a 5-point Likert-type scale (1 = never; 5 = always). A total score for empathy was computed through the mean over all items, with higher scores indicating higher degrees of empathy (consisting of empathic concern and perspective taking). Paulus (71) reported acceptable internal consistency for the German version (α = 0.71).

##### Collective moral norms

This construct reflects the dominating moral group norms and values, perceived by the group's members and was measured with the Collective Moral Attitude in Sport Groups scale [“Kollektiv-moralische Einstellung in Sportgruppen” (54)]. The scale consists of eight items that are answered on a 5-point Likert-type scale (1 = extremely; 5 = not at all). Example items are “In our training group, success is more important than dedication and loyalty” and “In our training group, assertiveness is more important than fairness”. The scale's score was computed by the mean, with higher scores representing high perceived moral group norms. The scale's internal consistency has shown to be very good (α = 0.91) and evidence for its construct validity is provided (54).

##### Pre- and post-intervention manipulation checks

In order to better understand and critically discuss the study results, athletes were asked to answer several questions, that differed from pre to post to follow up test. In the pretest the questions were “Have you participated in a doping prevention program/workshop before?” (yes/no) and “If yes, which one/what kind/what program (e.g., NADA workshop) was it?”. In the posttest the questions for the two intervention groups were “In how many of the six intervention sessions did you participate?” and “Which technical device did you use to participate in the Zoom sessions?” (laptop computer, tablet, smartphone, or other) whereas the questions for the control group were “Did you participate in any doping prevention program during the last 6–8 weeks?” (Yes/no) and “If yes, which one/what kind/what program (e.g., NADA workshop) was it?”. The same questions were used for the follow up measurement, with the modification of asking for “the last 3 months” instead of “the last 6–8 weeks”.

#### Procedure

This study was approved by the Ethics Committee of the authors' university. Study participants were recruited by contacting coaches or stakeholders of sport clubs and associations as well as teachers or youth officers of elite sport schools *via* telephone and email. They were provided with a brief study outline and asked for their interest in letting their athletes participate in the study. Teams, training groups, or classes that volunteered to participate were randomly assigned to one of the three conditions/groups by a research associate who was part of the research team. Randomization was performed by allocating teams/classes/training groups instead of individuals to avoid potential transfer effects of the differing contents of the interventions, that is, when individual athletes of one and the same team/class talk to each other about the contents of their respective intervention program. In order to ensure balance of athletes' gender and sport type across the three groups during the randomization process we applied minimization. This technique, which is seen methodologically equivalent to randomization (73), helped to minimize imbalances regarding important participant characteristics, i.e., gender and sport type, between the three groups.

Ahead of the first measurement point, participants and their parents/legal guardians received information about the study's aims and its voluntary nature, the warranty of treating all obtained information anonymously, and an outline of the investigation phase. After obtaining informed consent of the athletes (and their parents/legal guardians if athletes were under the age of 18), a Zoom link for the upcoming online sessions was sent *via* Email to the responsible person of the respective team or class who forwarded it to the participating athletes. The first online session in all three conditions in which the group comes together with the facilitator was the pretest. The athletes attended the sessions from a location they chose and, in general, used one device (e.g., laptop or tablet) per person. For the teams/classes that were assigned to either the values-based or the information-based intervention, their respective program started 1 week after the pretest and had a duration of 6 weeks with one 45-min synchronous online session per week. The teams/classes that were assigned to the control condition did not receive any intervention during that period of time. Six to eight weeks after the pretest athletes of all conditions came together with their respective group in their respective Zoom session for the posttest. For athletes of the values-based as well as the information-based intervention condition this was followed by a 3–4-month no-treatment phase and the follow up measurement in a final Zoom session. During these months, i.e., after completion of the interventions' implementation phase, the waiting control groups/teams were provided with the material of the values-based intervention program for ethical/equal treatment reasons.

For all measurement points participants joined a Zoom session, in which the instructor provided the link to an online questionnaire. This questionnaire consisted of sociodemographic questions (e.g., age, gender, sports, years competing, etc.), the measures described above, pre- and post-manipulation checks as well as an individual password, that the athletes created themselves. This password was used to connect the data, obtained at the two, respectively, three measurement points, while at the same time ensuring anonymity and, hence, reducing social desirability in athletes' responses.

All intervention sessions as well as data collection sessions were conducted by one of two trained facilitators who both were members of the research team. They were one female and one male facilitator both with extensive experience in instructing adolescent athletes, sport school students as well as university sport students. Since they substantially took part in the intervention development they were well acquainted with the material and intended delivery. Additional training for the instructors was ensured by having them conduct the sessions in the two pilot studies (one in person and one online), in which they were observed by colleagues from the research team and received feedback regarding their intervention implementation. By the time the interventions were adapted to an online format both instructors had undertaken various Zoom meetings both from a teacher's and participant's perspective and therefore were able to competently lead the sessions *via* Zoom. The instructors communicated with coaches and/or athletes in between the sessions and measurement points for scheduling/rescheduling Zoom sessions according to the teams'/classes' convenience and sent out reminders for each session and each measurement point in order to retain groups and athletes in the study. Additionally, before and in each measurement session, athletes were encouraged to fill in and complete the online questionnaire by outlining the importance of its completion in relation to the short amount of time needed to do so. Also they were reminded that, upon completion of the last measurement point, small thank-you gifts (e.g., colored pens) would be sent out to the participating clubs/schools. Study implementation occurred over a period of several months with individual starting points for each team/class depending on their annual training and competition schedule.

#### Data analysis

Data were analyzed with IBM SPSS Statistics 27.0. and R language 4.1, employing multilevel modeling. Specifically, general linear mixed models with adjustment for team/class level clustering as a random effect were performed to assess intervention effects for our outcomes (doping intention, doping susceptibility, moral disengagement, anticipated guilt, empathy, and collective moral norms). Data were collected and analyzed on the individual level (athletes, level 1) and athletes were nested in teams/classes (level 2). Research question 1 was analyzed by conducting two-level regressions with each of our outcomes at posttest as the dependent variable, adjusted for the respective pretest score and intervention group (i.e., condition). We also adjusted for gender, age, and exposure to external doping prevention measures. The intervention effect for each outcome represents the effect the condition had on the respective regression slope, compared to the control group. For research question 2, three-level regressions were conducted, with repeated measures outcomes (pre, post, follow up; level 1) nested in athletes (level 2) and athletes nested in teams/classes (level 3). Again, the models were adjusted for condition, gender, age, and exposure to external doping prevention measures.

For all analyses, confidence intervals (CI) were used for interpretation of the meaningfulness of changes in outcomes. This entails that when CIs do not include zero, an effect is classified as significant. If effects were found to be significant, effect size Hedge's g (for comparing to the control group) or Cohen's d (for comparing different time points within one condition) was computed and interpreted according to the following rules of thumb: 0.2 = small effect, 0.5 = medium effect, and 0.8 = large effect. All analyses were carried out per protocol which means that athletes who did not complete all measurement points for the selected analysis were listwise deleted. As suggested in the CONSORT Guidelines (73), we did not examine if there were significant group differences at baseline.

## Results

**Figure 1:**
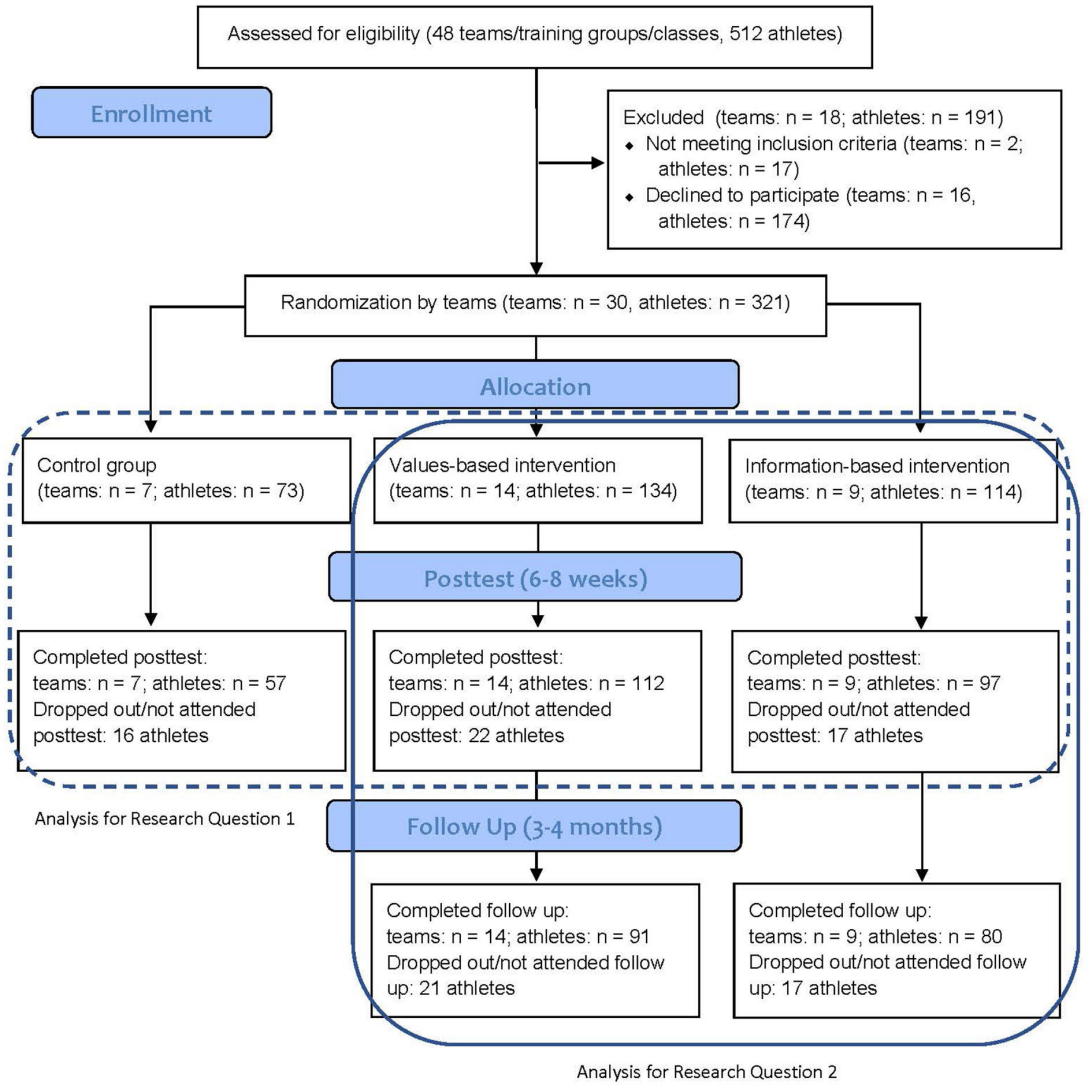
CONSORT participant flowchart.

A CONSORT flow diagram is depicted in Figure 1 and describes the flow of participants and teams/classes through the study's phases. Retention was satisfactory with a rate of 80% at posttest and 69% at follow up (for addressing research question 2, hence, without control group). The majority of athletes in the intervention conditions attended at least five intervention sessions (76.4%). Table 2 provides the descriptive statistics in the form of unadjusted means and standard deviations and corresponding sample sizes for all outcomes at pre, post and follow up assessments. The intervention effects for the comparison of the values-based and info-based condition with the control condition at posttest can be seen in Table 3. In contrast, Table 4 shows adjusted pre-post and post-follow up changes in outcomes for each of the two intervention groups.

**Table 2:** Unadjusted means (SD) for all outcomes by experimental group.

		**Values**			**Information**			**Control**		
		***N***	***M***	***SD***	***N***	***M***	***SD***	***N***	***M***	***SD***
Doping susceptibility	Pre	134	3.46	2.06	114	3.39	2.08	73	2.91	1.84
	Post	112	3.36	1.95	95	3.33	2.17	57	3.56	1.67
	Follow up	91	3.48	2.08	78	3.42	2.21	–	–	–
Doping intention	Pre	134	2.17	1.51	114	1.72	1.11	73	2.11	1.34
	Post	112	2.05	1.41	95	1.72	0.98	57	2.45	1.55
	Follow up	91	2.05	1.29	77	1.56	0.90	–	–	–
Moral disengagement	Pre	134	2.05	0.90	114	1.91	0.78	73	1.91	0.92
	Post	112	1.71	0.69	95	1.66	0.68	57	1.95	0.86
	Follow up	91	1.76	0.77	77	1.71	0.68	–	–	–
Anticipated guilt	Pre	134	5.79	1.40	114	6.06	1.23	73	6.05	1.20
	Post	112	6.16	0.99	95	6.13	1.33	57	5.93	1.46
	Follow up	91	5.34	0.87	77	6.02	1.32	–	–	–
Empathy	Pre	134	3.49	0.58	114	3.42	0.61	73	3.48	0.53
	Post	112	3.45	0.57	94	3.47	0.56	57	3.65	0.55
	Follow up	91	3.82	0.54	77	3.43	0.73	–	–	–
Collective moral norms	Pre	134	4.10	0.70	114	4.20	0.73	73	3.98	0.86
	Post	112	4.10	0.88	95	4.28	0.69	57	4.05	0.74
	Follow up	91	4.19	0.79	77	4.24	0.67	–	–	–

**Table 3:** Intervention effect for both interventions at posttest compared to control group.

	**Values**	**Information**	
	**Estimate (95% CI)**	**Estimate (95% CI)**	**ICC**
Doping susceptibility	−0.33 (−0.74, 0.08)	−0.35 (−0.77, 0.08)	0.01
Doping intention	−0.36 (−0.81, 0.09)	−0.42 (−0.91, 0.06)	0.12
Moral disengagement	−0.52 (−1.02, −0.03)	−0.51 (−1.04, 0.03)	0.10
Anticipated guilt	0.59 (0.13, 1.06)	0.48 (−0.01, 0.97)	0.08
Empathy	−0.10 (−0.37, 0.18)	−0.03 (−0,32, 0.26)	0.001
Collective moral norms	−0.03 (−0.34, 0.26)	0.002 (−0.31, 0.31)	0.005

Estimate = effect of experimental condition on outcomes; gray shade = CI excludes zero.

**Table 4:** Adjusted pre-post and post-follow up changes by intervention groups.

	**Values**	**Information**
	**Estimate (95% CI)**	**Estimate (95% CI)**
**Doping susceptibility**
Pre-post	−0.04 (0.22, −0.29)	−0.06 (0.20, −0.33)
Post-follow up	0.10 (−0.17, 0.38)	0.04 (−0.26, 0.35)
**Doping intention**
Pre-post	−1.2 (0.12, −0.35)	−0.06 (0.20, −0.31)
Post-follow up	0.04 (−0.22, 0.30)	−0.18 (−0.47, 0.11)
**Moral disengagement**
Pre-post	−0.38 (−0.14, −0.61)	−0.31 (−0.05, −0.57)
Post-follow up	0.08 (−0.18, 0.34)	0.10 (−0.20, 0.40)
**Anticipated guilt**
Pre-post	0.26 (0.48, 0.03)	0.001 (0.27, −0.26)
Post-follow up	−0.74 (−0.99, −0.49)	−0.07 (−0.38, 0.24)
**Empathy**
Pre-post	−0.05 (0.17, −0.27)	0.15 (0.45, −0.15)
Post-follow up	0.93 (0.69, 1.18)	−0.04 (−0.37, 0.28)
**Collective moral norms**
Pre-post	0.02 (0.25, −0.21)	0.18 (0.43, −0.07)
Post-follow up	0.11 (−0.15, 0.36)	−0.10 (−0.40, 0.19)

Estimate = changes in outcomes; gray shade = CI excludes zero.

### Results for research question 1

As shown in Table 3, athletes in the values-based intervention reported a stronger decrease in moral disengagement directly after the intervention (i.e., at posttest), compared to the control group [−0.52, 95% CI = −1.02, −0.03, effect size (ES) Hedge's g = −0.43]. Furthermore, a significant effect was found for anticipated guilt. Athletes in the values-based intervention showed a greater increase in anticipated guilt at posttest (0.59, 95% CI = 0.13, 1.06, ES Hedge's g = 0.57) compared to the control group. No immediate intervention effects for other outcomes were found for the values-based intervention, nor for the info-based intervention.

### Results for research question 2

There was a significant reduction in moral disengagement from pre to post intervention for athletes in the values-based condition (−0.38, 95% CI = −0.14, −0.61, ES Cohen's d = −0.42) and for athletes in the information-based condition (−0.31, 95% CI = −0.05, −0.57, ES Cohen's d = −0.34) (please see Table 4). For both groups there were no further changes in moral disengagement from post to follow up, indicating that the reduced moral disengagement was sustained also 3–4 months after the intervention had ended.

A significant increase in anticipated guilt occurred from pre- to posttest but only for the values-based group (0.26, 95% CI = 0.48, 0.03, ES Cohen's d = 0.31). From posttest to follow up test, however, athletes in the values-based intervention could not maintain this increase in anticipated guilt, but, instead, reported a significant reduction in anticipating guilt, compared to the posttest (−0.74, 95% CI = −0.99, −0.49, ES Cohen's d = −0.66).

For empathy, no effects emerged from pre to post in both groups, though, athletes in the values-based group reported increased empathy from post to follow up (0.93, 95% CI = 0.69, 1.18, ES Cohen's d = 0.56) indicating that there was no immediate effect (posttest), but a potential “delayed” effect of the values-based intervention on empathy.

## Discussion

As doping has detrimental consequences for athletes and the integrity of sports, the need for effective prevention programs is obvious. Primary prevention through education with a focus on values, emotions and morality is seen as a promising approach to minimize doping (7) and research indicates which variables exactly could be addressed in anti-doping efforts. This study presents the development, implementation, and evaluation of a values-based anti-doping intervention that focuses on variables that have been empirically associated to doping intention, likelihood, or behavior [e.g., (33, 39)].

### Effects on outcomes

Our first research question addressed the immediate (post intervention) effects of our values-based intervention and the information-based intervention in comparison with a no-intervention waiting control group. In line with our expectations, the values-based intervention, compared to a control group, successfully decreased moral disengagement and increased anticipated guilt at posttest, whereas no changes in those variables emerged in the information-based intervention, when compared with a control group. This supports existing literature [e.g., (31)] and highlights the importance of going beyond mere knowledge provision in prevention efforts. Contrary to our expectations we did not find a significant immediate effect for doping intentions, doping susceptibility, empathy, and collective moral norms. Concerning the latter, we can conclude that collective moral norms that usually develop over time could not be increased in only one session dedicated to this topic and in a relative short amount of time. However, since social context variables are essential in forming a person's (anti-) doping attitudes and intentions (33, 34, 54), it seems worthwhile to target this variable more intensively and examine the dosage and time scope needed to produce changes. Most likely, programs aiming at entire teams/clubs and which also involve the coach might have a greater impact on this variable than our intervention which mainly addressed the individual athlete.

Even though the effects of the values-based intervention on doping intentions and susceptibility were not significant at the 5% level (95% CI), the estimates and CIs in Table 3 show the trend that both variables slightly decreased. It can be assumed that if the intervention had been more intense and had lasted longer, significant changes might have occurred.

Unfortunately, we were not able to make comparisons with a control group at follow up since for ethical reasons the control group received the values-based material shortly after the implementation had ended (it was not feasible for them to wait 3–4 months). Therefore, for the direct comparison with an untreated control group as reference group no conclusions about the effects' sustainability can be drawn. This is a methodological limitation that needs to be considered when planning and designing future studies.

Nevertheless, we were able to gain insight into how the slopes of each intervention group developed over time from pre to post, and post to follow up, as we collected data of both intervention groups at a third measurement point (research question 2). Results show that moral disengagement decreased from pre to post in both intervention groups, but not from post to follow up, demonstrating that the effects are maintained over time (i.e., 3–4 months after completion of the interventions). For anticipated guilt, an effect was found only for the values-based intervention at posttest, indicating that, for direct effects, this way of intervening might be more effective on outcomes that involve emotions, as compared to information provision. Surprisingly though, this effect was not maintained from post to follow up, but instead, anticipated guilt decreased, implying that the intervention effects on anticipated guilt could not be maintained over time. This could indicate a need for regular exposure to prevention efforts in order to ensure that effects are sustainable, but of course, the question of feasibility also has to be considered. Interestingly, for empathy, the effect was the other way around, namely there was no immediate effect at posttest, compared to pretest in both conditions; but at follow up athletes in the values-based intervention showed an increase in empathy. This finding suggests, that some effects might occur with a delay.

### Limitations, benefits, and future research directions

In our effort to compare three conditions, we must consider that the facilitators who conducted the two intervention programs and collected the data of all three groups were aware of the expected results, that is, in which condition/group changes in outcomes were expected. Therefore, experimenter bias could have occurred in the way that the researcher might have been more motivated when conducting the intervention program which is supposed to lead to changes in outcomes. We tried to minimize this bias by preparing educational materials that are not only appealing to participants but also to the instructors, thereby ensuring high levels of joy and motivation in teaching both intervention programs.

In analyzing the data we used a per protocol approach which means that athletes who did not complete all measurement points for the selected analysis were listwise deleted. This provides us with full records in our models, however the possibility of a “completers-only” bias (74) persists. Another factor to consider is that participants may have had different preconditions regarding their anti-doping education. For the majority of athletes, especially for national athletes, doping prevention education (e.g., NADA workshops) are compulsory at least once a year. However, for some athletes this might have been their first experience with doping prevention. Additionally, participants especially from the control group may have received other doping prevention measures during the study's investigation phase which could have resulted in performance bias. By including pre- and post-intervention manipulation checks in our model (e.g., “Have you participated in a doping prevention program/workshop before?” at pretest; Have you participated in any further doping prevention program/workshop since the beginning of this program?” at post and follow-up) we controlled for these factors. Results of our analyses showed that there was no significant impact of exposure to other doping prevention measures before or during the intervention on the outcomes. In future studies, an additional manipulation check of whether participants received general drug prevention programs outside of sport could be incorporated in order to control for its impact on the findings, too.

For the delivery mode of both interventions online teaching was chosen in order to conduct the programs independently of pandemic-related restrictions in face-to-face teaching. Although online learning/teaching entails several drawbacks, such as technical problems or insufficient technical skills, altered group dynamics, and information loss due to transmissions delays, there are multiple advantages that have to be highlighted. The synchronous online sessions enable participants and facilitators to independently choose their workplace, thereby saving time and reducing costs for getting to a specific location. We can conclude from the pilot studies that the online delivery mode even seemed to enhance the attractiveness of participation. In addition to the autonomy of choosing a location, athletes praised the versatile methods that were applied online, e.g., working with the interactive whiteboard, watching the animated dilemma video, or working together in breakout rooms, stating that this variety contributed to interesting and even entertaining sessions. Further support for online learning/teaching is provided by research in [e.g., (26)] and outside of sport science (75), demonstrating efficacy of online delivered interventions. Finally, as we targeted young individuals belonging to a generation that, in general, is used to a safe handling of contemporary media, online teaching seemed to be a useful and suitable approach.

In the current study the developed intervention programs are either values-based or information-based. The values-based program is devoid of any content of the information-based program and the other way around. This strict separation allows us to draw conclusions about each intervention's effectiveness in influencing doping-related variables and doping intention in comparison to each other and a no-intervention waiting control group. However, it is argued that doping prevention programs, in order to be effective in minimizing actual doping behavior, should be multifaceted and incorporate both values-based education and knowledge-transfer about doping (18, 58). This is in line with Woolf's [(8), p. 2] notion that “information does have to play a role promoting doping-free sport”, particularly to prevent unintentional doping. Thus, when delivering prevention programs in practice, it is recommendable to combine both approaches, for example, by adding an introduction session to the values-based program, in which comprehensive information about doping is conveyed (e.g., definition of doping, banned substances, health consequences, control system, etc.). Future research could evaluate if this combination of elements may be more effective in changing outcomes.

Concerning the process evaluation and evaluation of the specific contents of our values-based intervention, this study does not include any qualitative data. However, for both programs it would be valuable to gain insight into athletes' perception of a variety of issues (e.g., which activities the participants liked the most, which sessions or activities particularly provoked their critical thinking, how did they perceive participants' engagement). Therefore, in a subsequent study we plan to incorporate a mixed design that contains a qualitative approach. Nevertheless, at the present time, we can draw upon qualitative feedback from the pilot studies. According to athletes' responses in a feedback questionnaire after each session as well as within the general discussion after completion of the intervention, they seemed to view their participation as very beneficial. For instance, they stated to better be able to argue about the decision for or against doping, to have learned more about their and their groups' value priorities, to have reflected on their attitudes and feelings, or to have thought about the consequences someone's decision to dope has for others. These responses suggest that the values-based intervention, seen from a preliminary qualitative perspective, might indeed be a promising approach to encourage cognitive, emotional and group dynamic processes and to create awareness and reflection of personal values and attitudes in regard to doping.

### Practical implications

Since the German NADA is highly interested in incorporating values-based approaches into their educational material, workshops and website (and is already doing this) they are the main multiplier for the developed material as they will integrate elements of the values-based program into their material (e.g., activities to reduce moral disengagement). Since the intervention sessions were designed for 45 min in order to match the timeframe of a school lesson, an integration of topics (again, for example, the moral disengagement session) into the curriculum in elite sport schools, e.g., within PE or ethics lessons, is of particular interest. However, not only elite athletes should profit from doping prevention, but also athletes participating in grassroots sports could benefit from values-based anti-doping education [see (23)], which could also be incorporated in the general school setting [in line with (76)].

### Conclusion

Our study has shown, from a quantitative perspective, that the values-based intervention can produce desired changes in some, yet not all of the targeted outcomes. It represents a starting point for values-based anti-doping education within the German speaking countries, since it is the first large sample study to comprehensively develop, implement, and evaluate an intervention that targets moral variables empirically related to doping in a longitudinal experimental design. The values-based intervention program is grounded on theory, empirical evidence and preceding intervention projects from in and outside the German language area and is designed for young athletes, guided by pedagogical expertise. This study, therefore, responds to the call for values-based anti-doping education, emphasized by WADA's new International Standard for Education, and lays a foundation for subsequent research.

## Data availability statement

The raw data supporting the conclusions of this article will be made available by the authors, without undue reservation.

## Ethics statement

The studies involving human participants were reviewed and approved by Ethics Advisory Board of Leipzig University. Written informed consent to participate in this study was provided by the participants' legal guardian/next of kin.

## Author contributions

TM and AME conceptualized the study. NW and AME supervised the study and further conceptualized methodology with TM. KS and TM contributed to recruitment of participants, implementation of the study, and data collection. TM and TS analyzed the data. TM wrote the draft of the manuscript. NW and AME commented, edited, and reviewed it for its final version. All authors contributed to the article and approved the submitted version.

## Funding

The No2Doping project was funded by the Federal Institute for Sports Science (ZMVI4-070301/19-21). We acknowledge support from Leipzig University for Open Access Publishing.

## Conflict of interest

The authors declare that the research was conducted in the absence of any commercial or financial relationships that could be construed as a potential conflict of interest.

## Publisher's note

All claims expressed in this article are solely those of the authors and do not necessarily represent those of their affiliated organizations, or those of the publisher, the editors and the reviewers. Any product that may be evaluated in this article, or claim that may be made by its manufacturer, is not guaranteed or endorsed by the publisher.

## Footnotes

^1^Ethics is a subject that is taught in most German high schools just like PE and for which teachers need a university degree in ethics.

^2^Comprehensive materials for all sessions in both interventions can be obtained from the first author upon request.
